# Exploring the interactions between algae and archaea

**DOI:** 10.1007/s42995-024-00217-1

**Published:** 2024-04-08

**Authors:** Jie Lian, Dayu Zou, Lukas M. Trebuch, Changhai Duan, Meng Li

**Affiliations:** 1 College of Civil and Transportation Engineering, Shenzhen University, Shenzhen, 518060 China; 2 Archaeal Biology Center, Institute for Advanced Study, Shenzhen University, Shenzhen, 518060 China; 3 Shenzhen Key Laboratory of Marine Microbiome Engineering, Institute for Advanced Study, Shenzhen University, Shenzhen, 518060 China; 4 Department of Aquatic Ecology, Netherlands Institute of Ecology (NIOO-KNAW), Droevendaalsesteeg 10, 6708 PB Wageningen, The Netherlands; 5 SZU-HKUST Joint PhD Program in Marine Environmental Science, Shenzhen University, Shenzhen, 518060 China; 6 Department of Ocean Science, The Hong Kong University of Science and Technology, Clear Water Bay, Hong Kong SAR China

**Keywords:** Algal–archaeal interactions, Biogeochemical cycles, Archaeal isolation, Algal biotechnology

## Abstract

Algae and archaea co-exist in diverse aquatic ecosystems and play a significant role in ecological functions and biogeochemical cycles. Compared to well-studied algal–bacterial interactions, there is a lack of information on algal–archaeal interactions and how their interactions affect their physiological fitness and nutrient cycles in either artificial cultivation systems or natural environments. The vast archaeal biodiversity, as indicated by genomic sequencing and computational approaches, has stimulated great interest in exploring uncultivated archaea to expand our knowledge of algae-archaea symbiosis. In this review, we summarize the latest studies on the diversity of algae-associated archaea and their (putative) symbiotic interactions, highlight the effects of algal–archaeal interactions on biogeochemical cycles and extend such knowledge to facilitate novel archaeal isolation and a broad range of algae-based biotechnological applications.

## Introduction

Algae play critical roles in the nutrient cycles of lakes, oceans, and the atmosphere (Falkowski et al. 2008; Smetacek and Cloern 2008). They have also received particular attention from bio-based economies as they comprise one of several sustainable alternatives to fossil fuels (Lian et al. 2018; Wijffels and Barbosa 2010). In addition to abiotic factors, including temperature, light and nutrients, algae are critically dependent upon interactions with co-occurring microbes for growth and survival (Amin et al. 2015; Seymour et al. 2017).

Archaea were previously believed to exclusively inhabit extreme environments such as hot springs and deep-sea hydrothermal vents (Woese et al. 1978). However, the discovery of mesophilic archaeal groups in temperate and oxygenated marine waters suggests that these microorganisms may be more widespread and ecologically important than previously thought (DeLong 1992; Fuhrman et al. 1992). Both archaea and bacteria are ubiquitous nutrient remineralizers. However, to date only bacteria are frequently reported as being associated with algae in natural and engineered aquatic systems. For instance, bacteria contribute to microalgal health through recycling and solubilizing necessary elements to bioavailable forms (Amin et al. 2009; Clarens et al. 2010), synthesis and release of vitamins (Croft et al. 2005), excretion of growth-promoting phytohormones (Amin et al. 2015) and inactivation of algal pathogens via antibiotics (Seyedsayamdost et al. 2011). By contrast, knowledge of algal–archaeal interactions is almost non-existent compared to bacterial counterparts.

Archaea, like bacteria, utilize dissolved organic matter and respond to signaling molecules released by algae (Amin et al. 2012). Ongoing encounters between these organisms influence the co-evolution and ecology of both taxa in diverse ways. First, archaea are increasingly accepted as the ancestor of eukaryotic algae through engulfing a cyanobacterium that was retained as an organelle to perform photosynthesis; this process is known as serial endosymbiosis (Yoon et al. 2004). Moreover, horizontal gene transfer from various bacteria and archaea to algae has expanded their metabolic flexibility and enabled an adaptation to extreme environments (Schönknecht et al. 2013). More recently, the rapid expansion of genomic data has led to an improved understanding of archaeal diversity, with increasing evidence supporting the positive correlation of certain archaeal taxa with algae based on 16S and 18S rRNA gene sequencing (Hamilton and Havig 2017; Needham et al. 2018; Needham and Fuhrman 2016). However, as Olson and Kellogg (2010) have noted, analyzing algal–archaeal interactions is hindered by the challenge of isolating archaea. Despite rapid methodological and technological advances, the successful culturing of novel archaeal representatives remains limited (Sun et al. 2020). It is essential to isolate and culture species from those uncultured archaeal lineages (such as Marine Group II and III) to establish algae-archaea co-culture models for better understanding their physiology and ecological roles.

Archaea and algae are involved in the cycling of essential elements such as carbon, nitrogen, oxygen, and sulfur, as well as other trace metals (Offre et al. 2013). Their complex interactions are potent to change the atmospheric pool of greenhouse gases such as CO_2_ and CH_4_ through carbon fixation and methanogenesis. Similarly, the metabolism of volatile dimethylsulfide, a chemical involved in cloud formation and climate regulation (Hatton et al. 2012), could be influenced by algae-archaea symbiosis. Investigating algae-archaea interactions provides a foundation for the proper management of aquatic ecosystems in the context of global climate change. Moreover, understanding such interactions has diverse biotechnological applications, such as adding beneficial archaea into algal cultures to improve algal biomass accumulation and reduce production cost, or utilizing methanogenic archaea in biogas production from algae residual biomass. Proteins or secondary metabolites produced by archaea also have potential as biological agents in algal biomass harvest and cell disruption prior to biorefinery. The aim of this review is to provide both an overview of algal–archaeal interactions and also new perspectives on how to utilize such knowledge in algal biotechnology.

## Co-occurring archaea with algae

### Macroalgae associated archaea

The macroalgal surface is an ideal habitat for microbiota due to the high organic carbon content and abundant oxygen, and nutrients (Martin et al. 2014; Zulkifly et al. 2012). It has been reported that epiphytic microbial communities on macroalgae are usually distinct from that of the surrounding environment, which indicates that selection processes together with stochastic recruitment of microbes drive macroalgal surface colonization. The associated microbial communities (including archaea, bacteria, fungi, microalgae, protozoa, and viruses) together with the algal host are increasingly regarded as a functional unity called a holobiont. The highly specialized symbiotic interactions between all the involved organisms are important for the fitness of the host and for the functioning of the holobiont (van der Loos et al. 2019).

A large and growing number of microbial-macroalgal studies focus on bacteria (Zulkifly et al. 2012). For instance, it has been found that the brown macroalga *Ectocarpus* sp. strain 371 when deprived of associated bacteria were unable to survive a salinity change, while this capability could be restored by restoring their associated bacteria (Dittami et al. 2016). Bacteria are also implicated in the morphogenesis of the macroalga as evidenced by an abnormal tissue development of *Ulva mutabilis* under axenic conditions, whereas the alga completely recovers morphogenesis when cocultured with *Roseovarius* sp. and *Maribacter* sp. (Alsufyani et al. 2020).

Although metagenomic research suggests that bacteria dominate macroalgal-associated microbial community (de Oliveira et al. 2012), other members of the microbial community are also indispensable components of the holobiont. Archaea, as ubiquitously distributed taxa in diverse environments, are among important members of macroalgal epiphytic communities, but very little is known about their diversity and relationships between the macroalgal hosts and archaea (van der Loos et al. 2019). In many cases, archaeal taxa are often excluded from downstream analysis of macroalgal-associated microbiome due to their low abundance and lack of diversity (Bengtsson et al. 2012).

Based on the limited number of studies, most macroalgal-associated archaea have been found to belong to Nitrososphaeria (previously Thaumarchaeota) and methanogenesis Euryarchaeota (Table 1). Trias et al. (2012) evaluated ammonium-oxidizing archaea on three macroalgal species (*Osmundaria volubilis*, *Phyllophora crispa* and *Laminaria rodriguezii*). However, unlike other marine habitats, archaea were underrepresented compared with ammonium-oxidizing bacteria. Illumina sequencing of 16S rRNA genes of microbiota associated with *Sargassum* and *Ulva prolifera* has revealed the presence of several methanogenesis Euryarchaeota assigned to Methanomicrobiaceae, Methanosarcinaceae and Methanococcaceae (Hervé et al. 2021; Zhao et al. 2022). In addition to Nitrososphaeria and Euryarchaeota, a few other archaeal lineages are also found to co-exist with macroalgae. For instance, Nanoarchaeales (previously Nanoarchaota) and Woesearchaeales (previously Woesearchaeota) were associated with epilithic macroalgae (Titioatchasai et al. 2023), while Bathyarchaeia, Lokiarchaeia and Woesearchaeales were found during a green tide of *Ulva prolifera* (Zhao et al. 2022).

**Table 1:** Co-occurring archaea with macroalgae and microalgae

Algae specie	Co-occurring archaea	Sample region	References
*Macroalgae*			
*Osmundaria volubilis*, *Phyllophora crispa* and *Laminaria rodriguezii*	Nitrososphaeria	Shelf of Majorca and Minorca Islands	Trias et al. (2012)
*Sargassum*	Methanomicrobiaceae, Methanosarcinaceae, Methanococcaceae	Atlantic coasts of Martinique and Guadeloupe	Hervé et al. (2021)
*Ulva prolifera*	Marine Group II, Nitrosopumilaceae, unclassified Bathyarchaeia, Nitrososphaeraceae, Methanosarcinales, unclassified Thermoplasmata	Coastal Qingdao, China	Zhao et al. (2022)
*Padina* sp., *Lobophora variegata*, *Sargassum*, *Turbinaria*, *Cladophora* and red turf algae	Nanoarchaeales, Woesearchaeales	Reef crest at Ko Taen, Mu Ko Thale Tai National Park, the Gulf of Thailand	Titioatchasai et al. (2023)
*Pyropia haitanensis*	Nitrosopumilaceae	Rizhao City and Ningde City, China	Wang et al. (2022)
*Microalgae*			
*Bathycoccus prasinos*	Marine Group I, Marine Group II	San Pedro Ocean Time-series station	Parada and Fuhrman (2017)
Dinophyta, Chlorophyta, Bacillariophyta	Marine Group II	Tara Oceans expedition	Lima-Mendez et al. (2015)
*Phaeocystis*, *Chaetoceros*, *Heterosigma*	Marine Group II	San Pedro Ocean Time-series station	Needham and Fuhrman (2016)
Diatoms, *Pseudo-nitzschia*, *Chaetoceros*	Marine Group II	Offshore of Santa Catalina Island, California, USA	Needham et al. (2018)
*Micromonas pusilla*	Marine Group II	California Cooperative Fisheries Investigations Line 67	Orsi et al. (2015, 2016)
*Alexandrium catenella*	Marine Group II, Methanomicrobiales, Methanocellales, Halobacteriaceae, Thermoplasmatales	Salt Pond in the Nauset Marsh System on Cape Cod, USA	Zhou et al. (2018)
*Phaeocystis antarctica*	Marine Group I, Marine Group II, Marine Group III	Amundsen Sea	Kim et al. (2014)
Diatoms, green algae, haptophytes, stramenopiles	Marine Group II	Pearl River plume and the northern South China Sea	Chen et al. (2022)
*Phaeocystis*, *Rhizosolenia*, dinoflagellate	*Halobacterium*, Thermoplasmatales, Marine Group I, Marine Group II	German Bight	Wemheuer et al. (2012)
Bacillariophyta, Chlorophyta, Dinophyceae	Marine Group II	Pearl River, China	Xie et al. (2018)
*Chlorella vulgaris*	Haloarchaea, *Methanococcus*	Outdoor, alkaliphilic raceway pond	Bell et al. (2016)
*Coleochaete pulvinata*	*Methanosaeta*	Sharon Lake, Marquette County, Wisconsin	Knack et al. (2015)
*Chlamydomonas*, *Chloromonas*	Nitrososphaeria, Thermoplasmata	Mount Adams in Washington, and Mount Hood and North Sister in Oregon	Hamilton and Havig (2017)
*Noctiluca scintillans*	Methanocellaceae, Methanomicrobiaceae, Methanocorpusculaceae, Nitrosocaldaceae, Nitrosopumilaceae, Halobacteriaceae, Cenarchaeum	Coastal area of Dongchong in Shenzhen, China	Zhou et al. (2020)
*Dunaliella salina*	*Halobacterium*	Saltpans along the south eastern coast, Andhra Pradesh, India	Keerthi et al. (2018)

### Microalgae associated archaea

There is increasing evidence to suggest that microscale interactions play out within the region immediately surrounding individual microalgal cells, called the phycosphere (Seymour et al. 2017). The interactions between microalgae and microbes not only strongly affect carbon and nutrient cycling, regulate the primary productivity and stability of aquatic food webs, and affect ocean–atmosphere fluxes of climatically relevant gases (Amin et al. 2015), but also could have relevant industrial applications to increase algal biomass yield (Lian et al. 2018). Therefore, an increasing number of studies have assessed the microbial community associated with microalgae in a wide range of environments, the vast majority of which focus on bacteria. It has been shown from frequent observations that microalgae require bacteria for morphology development and growth (Bolch et al. 2011; Windler et al. 2014). Bacteria are able to fix nitrogen (Foster et al. 2011; Thompson et al. 2012) and synthesize an array of molecules, including vitamins (Grant et al. 2014; Xie et al. 2013), the growth-promoting hormone indole acetic acid (IAA) (Amin et al. 2015; Dao et al. 2018) and the siderophore vibrioferrin (Amin et al. 2007; Lupette et al. 2016) for microalgae to exchange for dissolved organic matter.

Like macroalgae-associated archaea, archaea co-occurring with microalgae have often been overlooked. By assessing the limited literature, marine group I (MGI, Nitrosopumilaceae) and marine group II (MGII, Poseidoniales) are found to be the most common archaeal group correlated with microalgae (Table 1). As the main archaeal taxa distributed in the global ocean, MGI and MGII account for 20% of the prokaryotic biomass in marine waters (Karner et al. 2001). MGI were generally negatively correlated with phytoplankton communities due to competition for ammonium (Liu et al. 2018; Murray et al. 1998), although positive correlations between MGI and phytoplankton populations have also been recorded in a few studies (Tolar et al. 2013; Wells et al. 2006). Observations at the San Pedro Ocean Time-series (SPOT) station demonstrated that MGII was positively correlated to a green microalga (*Bathycoccus prasinos*) and to another bloom-forming microalga *Phaeocystis globosa*, which potentially indicates that organic substrates from phytoplankton promote MGII heterotrophic growth (Needham and Fuhrman 2016; Parada and Fuhrman 2017). Changes in archaeal community composition have also been reported in blooms of *Phaeocystis antarctica* and most of the archaeal sequence reads could be classified into MGI, MGII and marine group III (MGIII) (Kim et al. 2014). Moreover, co-occurrence network analyses from the Tara Oceans expedition showed that MGII co-exists with a variety of phytoplankton including Dinophyta, Chlorophyta and Bacillariophyta (Lima-Mendez et al. 2015).

Members of methanogens and Thermoplasmatales are among the second most common archaeal group occurring with microalgae. For instance, during a spring bloom of the dinoflagellate *Alexandrium catenella*, archaea accounted for about 6%–10% of the total prokaryotic community, with the most abundant OTUs belonging to methanogens (Methanomicrobiaceae, Methanocorpusculaceae, Methanoregullaceae, and Methanocellaceae), ammonia-oxidizing archaea (AOA) (Nitrosopumilaceae and Nitrosocaldaceae), Halobacteriaceae and Thermoplasmatales (Zhou et al. 2018). Similarly, Methanogens and/or Thermoplasmatales co-existed with *Alexandrium catenella*, *Chlamydomonas* sp., *Chloromonas* sp., *Chlorella vulgaris* and *Noctiluca scintillans* (Bell et al. 2016; Hamilton and Havig 2017; Zhou et al. 2018, 2020). In alkaliphilic pond and saltpans, halophilic archaea were found to be present with *Chlorella vulgaris* and *Dunaliella salina* (Bell et al. 2016; Keerthi et al. 2018)*.*

### Growth-promoting archaea

**Fig. 1:**
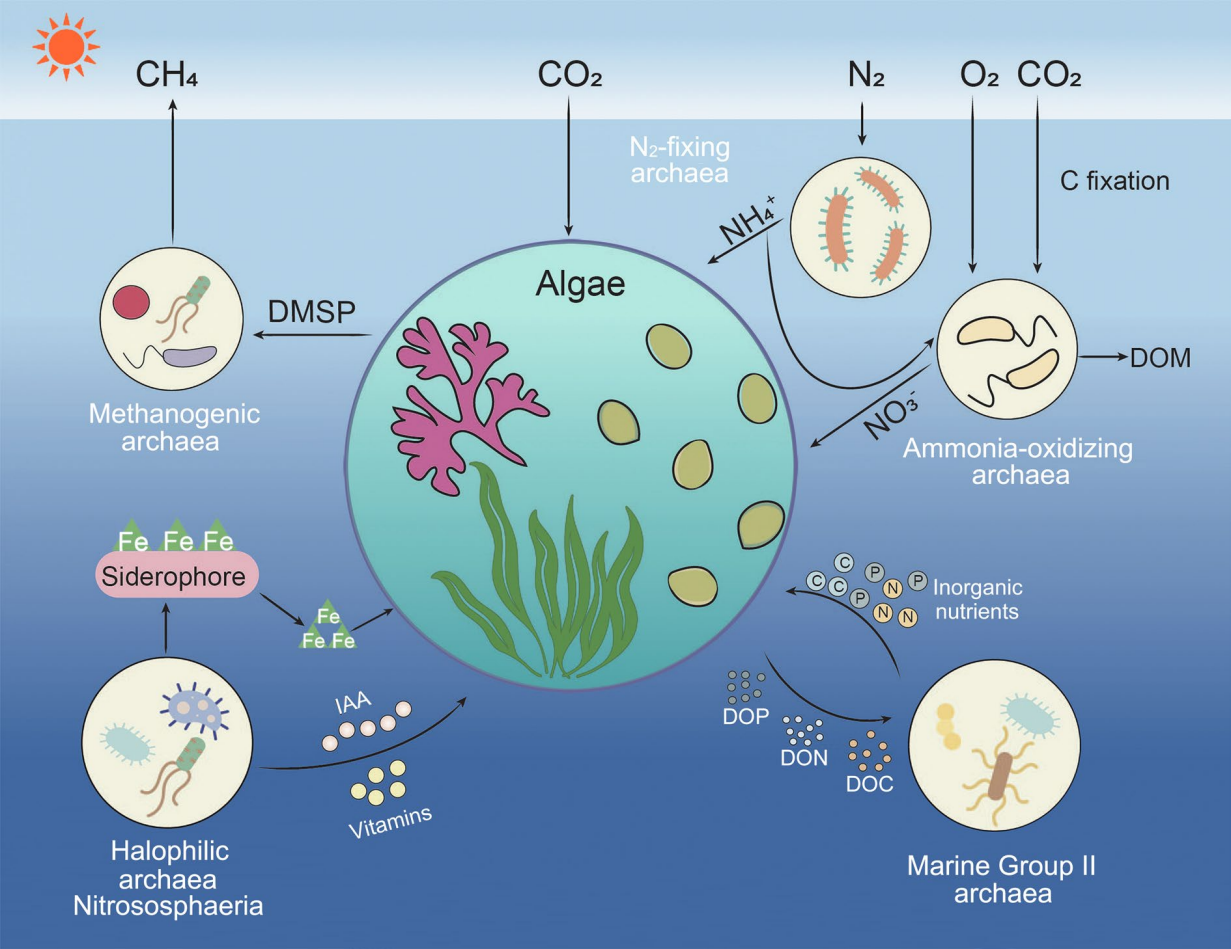
A simplified illustration of interactions between algae and archaea. Algae exude dissolved organic carbon (DOC), dissolved organic nitrogen (DON) and dissolved organic phosphorus (DOP) to archaea. In return, the archaea fix nitrogen and provide dissolved inorganic carbon (DIC), dissolved inorganic nitrogen (DIN), dissolved inorganic phosphorus (DIP) to support the growth of the algae. Archaea can also discharge methane through the metabolism of dimethylsulfoniopropionate (DMSP). In addition, the archaea supply vitamins as organic cofactors or produce indole acetic acid (IAA) and siderophores to make iron bioavailable to promote algal growth

Interactions between algae and archaea are typically inferred from bioinformatic and statistical analyses (Fig. 1), as experimental evidence is relatively scarce in the literature. To date, only one in vitro study has been reported on interactions between microalgae and archaea. In this study, co-culturing the microalga *Dunaliella salina* with archaeon *Halobacterium salinarum* was found to improve the algae’s capacity for carbon fixation. The possible explanation for this is that the dead algal cells release byproducts of photosynthetic carbon into the surrounding media. The byproducts are further metabolized and remineralized by archaea into a form that is readily consumed by the algae. In addition, adding the supernatant of *D*. *salina* culture into artificial seawater supported the growth of *H*. *salinarum* (Baliga 2015). Beneficial effects are more widespread in algal–bacterial interactions. For example, Samo et al. (2018) found enhanced carbon fixation in *Phaeodactylum tricornutum* when this alga was associated with the filamentous bacterium of *Haliscomenobacter* sp. The possible reason for this is that the attached bacteria respired the algal carbon and relieved microscale CO_2_ limitation in *P*. *tricornutum* while removing O_2_ from the microenvironment. In the meanwhile, *P*. *tricornutum* may have benefitted from cross feeding of ammonium by bacteria. However, *Haliscomenobacter* sp. incorporated more algal derived carbon than other attached bacterial cells.

Currently, most studies on the interactions between algae and associated archaea are limited to identifying correlations. These correlations can serve as a valuable starting point for developing hypotheses on functional relationships between algae and archaea but more experimental models of algal–archaeal interactions are needed to establish a guide for future research and a better understanding of the roles played by archaea in algal–microbial systems. The archaea on the surface of macroalgae or associated with bloom-forming phytoplankton (for instance, *Phaeocystis globose*) would be ideal targets to start with.

### Vitamin B_12_ provided by archaea

Based on a survey of 326 algal species, vitamin B_12_ (cobalamin) has been found to be an essential cofactor required for many branches of life, including more than half of microalgae (Croft et al. 2005), whereas the biosynthesis of vitamin B_12_ is confined to only a few bacteria and archaea taxa (Fang et al. 2017).

 Bacteria (*Mesorhizobium loti* and *Sinorhizobium meliloti*) are involved in algal symbiosis through cobalamin biosynthesis (Grant et al. 2014; Xie et al. 2013). However, the involvement of archaea in cobalamin production is poorly understood. Doxey et al. (2015) examined genomes and 430 metagenomes of Nitrososphaeria from a diverse range of aquatic environments and demonstrated that all analyzed genomes possess cobalamin synthesis genes. This result provides evidence that the growth of cobalamin-auxotrophic phytoplanktonic communities may depend on cobalamin-producing microbes such as Nitrososphaeria. Cobalamin-producing Nitrososphaeria could be an interesting candidate for isolation, and cobalamin exchange between algae and archaea needs to be validated in laboratory conditions.

### Phytohormone

Bacteria regulate algal growth by providing phytohormone (Amin et al. 2015; Dao et al. 2018), and archaea have a similar potential. In earlier research, archaea were found to produce large quantities of plant hormones. Specifically, the thermophilic archaeon *Sulfolobus acidocaldarius* produces the plant growth-stimulating hormone IAA at a concentration one thousand times greater than those found in typical plant extracts (White 1987). Recent metagenomic data analysis also suggests that archaea have the potential to produce plant growth-promoting auxin (Taffner et al. 2018). Hagagy et al. (2023) further confirmed that halophilic archaea are capable of synthesizing IAA. Considering the co-occurring history of halophilic archaea and algae (Table 1), it is probable that archaea are able to establish potentially mutualistic relationships with algae through the synthesis of phytohormones. The recognition of these interactions will have implications for the exploitation of algae for biotechnological applications.

### Siderophores

Iron is crucial for photosynthesis and respiration and its low solubility and concentration in parts of the ocean often limit primary productivity and the growth of bacteria (Amin et al. 2009). To overcome the limitation of iron, marine heterotrophic bacteria produce siderophores that strongly bind to iron and increase its solubility (Vraspir and Butler 2009). Microalgae do not synthesize siderophores. However, they are able to access iron from iron-complexed siderophores through involving a reduction step to release bound iron from the chelates (Hopkinson and Morel 2009).

Siderophores produced by bacteria have been extensively studied, for instance, *Marinobacter* sp., which lives in close association with *Scrippsiella trochoidea*, has been found to produce an unusual lower-affinity dicitrate siderophore that enhances iron assimilation by the alga (Amin et al. 2009). By comparison, siderophore production by archaea has received less attention (Barry and Challis 2009). Shafiee et al. (2019) found that the ammonia-oxidizing archaeon *Nitrosopumilus maritimus* was unable to produce siderophores, while a few halophilic archaea were capable of synthesizing siderophore (Dave et al. 2006; Hagagy et al. 2023). Halophilic archaea have been found to grow with algae (Table 1) and co-operation to scavenge the limiting micronutrient through the synthesis of siderophore is essential for the survival of both algae and archaea.

## Elemental cycles

### Carbon cycle

Microalgae and oxygenic phototrophic cyanobacteria are the dominant primary producers in aquatic ecosystems, responsible for nearly 50% of global photosynthesis (Field et al. 1998). Thaumarchaea in the ocean are also known to fix inorganic carbon, contributing approximately 1% to the total marine primary production (Könneke et al. 2014). These primary producers have complex metabolic interactions with the surrounding microbial community, which play critical roles in regulating the global carbon cycle (Kim et al. 2022). Although it is commonly believed that archaea benefit from the ready availability of organic carbon sources produced by the host alga, field data suggests that they are also able to take up organic carbon compounds (Ouverney and Fuhrman 2000; Teira et al. 2006) by expression of transporter proteins (Bergauer et al. 2018). For instance, MGII and MGIII archaea have been suggested to be heterotrophic, with a specialization in high-molecular-weight compounds (Zhang et al. 2015). More interestingly, most MGII taxa contain archaeal flagella gene operons used to attach to phytoplankton cells and crack intact phytoplankton cells to obtain organic matter (Lassak et al. 2012; Rinke et al. 2019). In addition, the cell density of MGII has been found to increase in the presence of alga-derived proteins or whole algal cells of *Micromonas pusilla* (Orsi et al. 2015, 2016), indicating the active uptake of organic substrates by archaea and potential interactions between two species. In hypersaline environments, it is suggested that photosynthetically produced glycerol leaking from the unicellular green algae *Dunaliella* sp. is the preferred carbon and energy source for the growth of halophilic archaea (Oren 1995).

### Nitrogen cycle

Marine nitrogen fixers (diazotrophs) play an irreplaceable role in marine ecosystems by converting atmospheric N_2_ gas to bioavailable nitrogen in the form of ammonium (Hutchins and Capone 2022). Although photoheterotrophic cyanobacteria are the most cosmopolitan diazotrophs found in a range of marine environments, *nifH* genes (nitrogenase) of non-cyanobacterial diazotrophs (NCDs) have also been detected (Farnelid et al. 2011). Archaeal methanogens are members of NCDs and make a major contribution to nitrogen fixation in the aphotic zone (Bombar et al. 2016). A survey on 1175 genomes of methanogenic Euryarchaeota found that 44 genomes have *NifHDKENB* genes (Koirala and Brözel 2021). Ammonium produced from nitrogen fixation is the preferred nitrogen source for algal growth because assimilation of ammonia is more energy efficient than other sources of nitrogen (Kong et al. 2021). Archaeal nitrogen fixation could thus have a strong impact on marine phytoplankton productivity, especially in oligotrophic waters. In addition, a wide range of studies have shown that heterotrophic diazotrophs are stimulated (increases in N_2_ fixation or *nifH* gene copy numbers) by nutrient or dissolved organic matter additions (Dekaezemacker et al. 2013; Loescher et al. 2014; Rahav et al. 2013). Hence, there are possible interactions between diazotrophic archaea and planktonic microalgae through exchange of fixed nitrogen and organic carbon.

Ammonia-oxidizing archaea (AOA), such as *Nitrosopumilus maritimus* SCM1, however, are ubiquitously distributed in marine and terrestrial environments (Walker et al. 2010) and play a significant role in the global nitrogen cycle, particularly in combination with anaerobic ammonium oxidation (anammox) and denitrification by bacteria (Francis et al. 2007; Pajares and Ramos 2019). AOA possess the capacity to both fix carbon by using inorganic carbon as the sole carbon source and also oxidize ammonia at extremely low concentrations (Könneke et al. 2005). Despite their high ammonia affinity and economical cell size, Thaumarchaea are apparently not as competent as phytoplankton at ammonia acquisition in some environments (Schleper and Nicol 2010; Wan et al. 2018). In addition, fast-growing phytoplankton, such as diatoms, that outcompete archaeal diazotrophs for other limiting nutrients are generally also considered to be poor competitors for acquiring phosphorous and iron (Ward et al. 2013). Therefore, competition for nitrogen and other nutrients makes interactions between phytoplankton, diazotrophic archaea and AOA more complicated.

### Phosphorus cycle

Phosphorus is an essential element of all lifeforms to synthesize DNA, RNA, ATP and the phospholipids of cell membranes (Ruttenberg 2001). The most abundant form of phosphorous in the global ocean exists in the + 5 oxidation state (phosphate) (Van Mooy et al. 2015). Phosphate availability can impact primary production rates in the ocean and in recent years it has been recognized that phosphate limitation may be more prevalent than previously thought (Paytan and McLaughlin 2007). Phytoplankton can utilize phosphate for growth and release a fraction of it as dissolved organic phosphorous (DOP). Archaea and bacteria synthesize alkaline phosphatases (Yadav et al. 2015), which hydrolyze DOP back to orthophosphate that can be reused by microalgae for further growth. Although algae also contain alkaline phosphatases to scavenge DOP under phosphate deficiency (Zhang et al. 2022), microbial phosphatases display higher efficiencies than those of algae. Indeed, previous studies have shown that halophilic archaea such as *Halobacterium* excrete different kinds of organic acids to lower pH and solubilize phosphate (Yadav et al. 2015). *Halobacterium* has been found in association with algae, especially under alkaliphilic conditions (Table 1). Further studies are needed to determine whether phosphate solubilizing archaea contribute to the growth of algae in phosphate-depleted environments.

### Sulfur cycle

Sulfate (SO_4_^2−^) is one of the most prevalent dissolved constituents in seawater, with a concentration of around 28 mmol/L (Andreae 1990). The high concentration of SO_4_^2−^ in seawater can readily be reduced by phytoplankton to synthesize a range of biomolecules. These phytoplankton-derived sulfur metabolites are released into seawater through exudation and cell lysis, which can be rapidly assimilated by marine bacteria and archaea as substrates or for the synthesis of vitamins, cofactors, signaling compounds and antibiotics (Moran and Durham 2019). The sulfur in these organic compounds is subsequently returned to sulfate by bacterial and archaeal catabolism, which can be reused by phytoplankton.

The sulfur-rich compound dimethylsulfoniopropionate (DMSP) is the best-studied marine sulfur metabolite. DMSP is an osmolyte that is mainly produced by marine algae in the photic zone and by bacteria in the aphotic zone and sediment (Zheng et al. 2020). Through grazing and/or virus-induced lysis, DMSP is released into the environment and later catabolized by diverse bacteria and phytoplankton via diverse DMSP lyase enzymes to generate the volatile dimethylsulfide (DMS). The DMS can then be further converted to dimethylsulphoxide (DMSO) or sulphate aerosols by bacteria (Curson et al. 2011, 2017). Methanogenic archaea have been shown to metabolize DMSP for the production of CH_4_ (van der Maarel and Hansen 1997). Zindler et al. (2013) measured concentrations of DMSP, DMS, DMSO and CH_4_, as well as various phytoplankton marker pigments in the surface ocean from Japan to Australia, and found positive correlations between DMSO and DMSP with chlorophyll a. In addition, CH_4_ production was positively correlated with DMSP and DMSO along the transect, which suggests that archaea feed on algae-derived DMSP for CH_4_ production. DMSP-dependent accumulation of CH_4_ has also been detected in the surface oligotrophic ocean as well as in phytoplankton blooms (Damm et al. 2010), lending further support to the notion that algae and methanogens have close associations.

DMS plays a role in cloud formation and potentially climate regulation (Todd et al. 2007; Zhang et al. 2019). CH_4_ is the second-most important greenhouse gas in addition to CO_2_ and accounts for 16–25% of atmospheric warming to date (Etminan et al. 2016). Moreover, Rosentreter et al. (2021) indicated that aquatic ecosystems contribute nearly half of total global CH_4_ emissions. Therefore, it is reasonable to speculate that algal–archaeal interactions could have a profound influence on global environmental change. Under global warming and ocean acidification conditions, both algal and archaeal communities can undergo major perturbations, further affecting DMSP metabolism and CH_4_ production. Consequently, unravelling interactions between alga and methanogenic archaea could not only help to deepen the understanding of the carbon and sulfur cycle but also help to provide better estimates of DMSP and CH_4_ production in oceans in the future.

### Application based on knowledge of algal–archaeal interactions

**Fig. 2:**
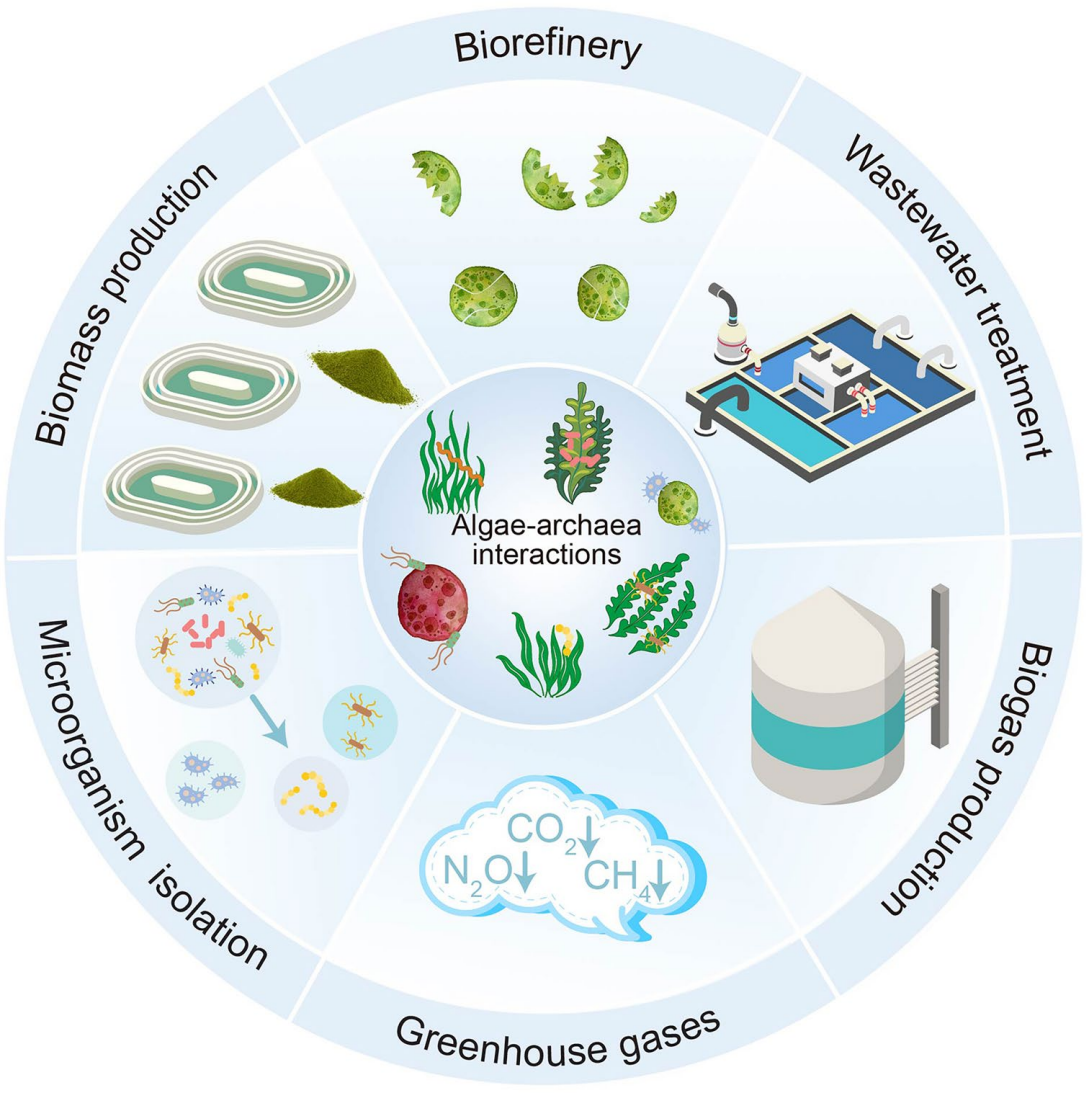
Potential application based on knowledge of algae-archaea interactions. Growth-promoting archaea can be used to stimulate biomass production of algae. Archaea-derived enzymes have the potential to disrupt algal cells prior to biorefinery. Algal–archaeal interactions have implications to improve the efficiency of algal-based wastewater treatment and biogas production. In addition, such knowledge can be implemented in algal-associated archaea isolation and mitigation of greenhouse gases emission

Elucidating interactions between algae and archaea will not only enrich the understanding of the full range of symbiosis on our planet, but also potentially provide a wide range of biotechnological and ecological applications (Fig. 2). Potential applications are described in the sections below.

### Living algae as a medium for archaeal isolation

A range of algae-associated archaea (MGII, for instance) are highly abundant in their habitats and likely play an important role in the biogeochemistry of that environment (Zhang et al. 2015). More importantly, currently, there are no cultured representatives of these archaeal clades, which makes them interesting to culture to better understand their functions (Lewis et al. 2021; Sun et al. 2020). Although numerous innovative methods, including reverse genomics (Cross et al. 2019), Raman-activated cell sorting (Lau et al. 2008), live-FISH (Batani et al. 2019), iChip (Nichols et al. 2010), SlipChip (Ma et al. 2014) and nanoporous microscale microbial incubators (Ge et al. 2016), have been adopted to isolate desired cells, the success remains limited. One reason for the failure of these methods could be the inability to provide suitable media that contains all of the necessary growth factors present in natural habitats (Kaeberlein et al. 2002).

**Fig. 3:**
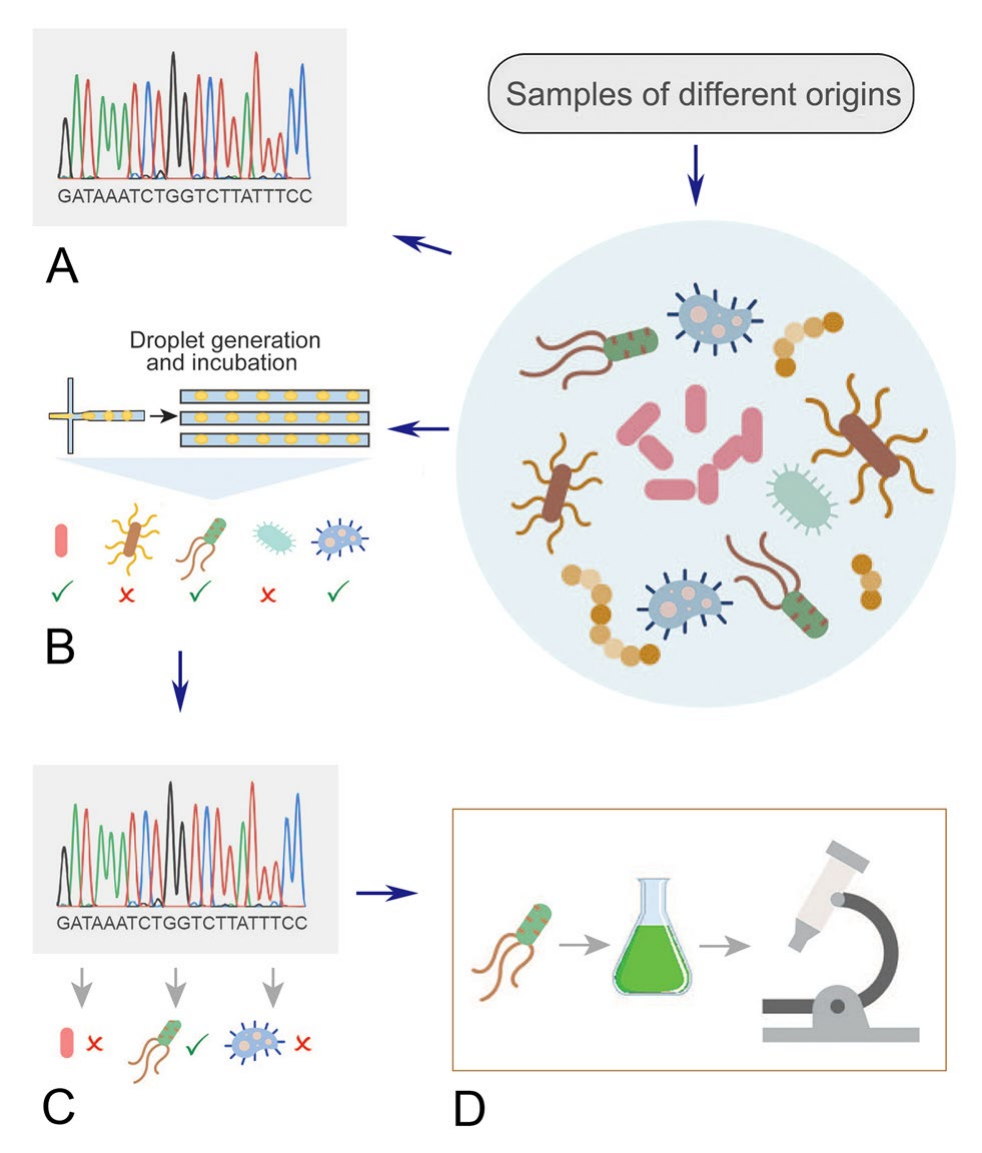
Schematic illustration of workflows for isolating novel archaea for cultivation using living algae as a medium. **A** Samples of various habitats can first be sequenced to identify target organisms of high relative abundance and their associated algae. **B** Microfluidic device can be used to generate droplets with single cell of prokaryotic microbes. Axenic algal cultures are prepared and inoculated into droplets to establish large numbers of cocultures. The cocultures were incubated and then screened for growth. **C** Viable cultures are sequenced for screening archaeal species of interest. **D** Cultured isolates can be used for downstream characterization and experimentation to investigate their physiology

Previously, information obtained from quantitative and qualitative analyses of photosynthetic metabolites of microalga (*Chlorella sorokiniana*) was used to formulate a novel artificial medium for the selective isolation of algal-associated bacteria (Watanabe et al. 2008). A small number of uncultured archaea are frequently found co-existing with algae. Exchange of essential growth factors between them has occurred to ensure the survival and success of both species, which can then be harnessed for the isolation and cultivation of novel archaea (Sun et al. 2020). Based on these developments, a method for isolating novel species using algal-microbial cocultures is proposed (Fig. 3). Through screening diverse habitats of archaea (algal bloom samples, algae-inhibited alkaliphilic ponds, etc.), the presence of target archaea for isolation and their possible algal symbionts can be determined (Fig. 3A). Then, the algal partner can be purchased (if possible) or manually isolated, and antibiotic treatment can be applied to prepare axenic cultures of algae. Single cells of microorganisms prepared from serial dilution or microfluidic systems can be inoculated into algal cells-containing microcompartments to establish cocultures. To increase scalability and throughput, microfluidic systems are preferred to encapsulate large numbers of single cell in droplets in parallel. Droplet containing axenic algal cells can be injected into droplets encapsulated with a single prokaryotic cell to establish cocultures. These droplets are incubated in the microfluidic device and can also be used to screen a range of different growth conditions (Fig. 3B). In this way, prokaryotic microbes rely on algae for essential growth factors instead of receiving excessive quantities of nutrients typically provided by classical media. The growth of microbes in cocultures can be routinely checked by sensors (optical density, fluorescence, etc.) or visualized by microscopic observation. The actively growing candidates can then be subjected to PCR with universal archaeal 16S rRNA gene primers and sequencing-based screening for taxonomic discrimination (Fig. 3C). Isolates of interest can be used for downstream characterization and experimentation to investigate their cell biology and physiology and to properly understand their ecological roles (Fig. 3D). More importantly, could these novel lineages interact with algae and how?

## Biotechnological application

### Symbiotic archaea to promote the growth of algae

Increasing evidence has confirmed that growing microalgae with beneficial bacteria contributes to an increase in photosynthetic capacity (Martin et al. 2021), carbon fixation (Samo et al. 2018), biomass yield and lipid synthesis (Berthold et al. 2019; Toyama et al. 2019), production of biohydrogen (Lakatos et al. 2014) and production of extracellular polymeric substances (Roux et al. 2021). Likewise, the growth-promoting effects of archaea have been verified in plants through the synthesis of IAA and siderophores, nitrogen fixation (Leigh 2000), phosphorus solubilization (Yadav et al. 2015), enhanced ammonia oxidation, nutrient recycling and the boosted defense system against pathogens (Song et al. 2019) to benefit the growth of plants. Undoubtedly, algae-growth-promoting archaea exist even though little evidence has been reported. Co-culturing of archaea and algae has great potential to enhance algal growth rates, prevent algal pathogens and reduce the production costs. Future research can be directed to find more growth-promoting archaea and to elucidate the molecular basis of algae-archaea interactions for large-scale biotechnological applications (Fig. 2).

### Downstream processing of algal biomass

Algal cell walls are comprised of a wide diversity of complex polysaccharides that are highly recalcitrant to cell disruption. Through complex and numerous interactions with the host, algae-associated microbes constitute a large source of bioactive compounds and specific polysaccharidases that are potentially useful in the downstream processing of algal biomass (Martin et al. 2014). Metagenomic analyses have shown that MGII archaea can express chitinase, glycoside hydrolase and protease to lyse algal cells for the acquisition of organic carbon (Damashek et al. 2021; Martin-Cuadrado et al. 2015; Rinke et al. 2019). Additionally, extremophilic archaea living in extreme environmental conditions have developed enzymes with increased stability at high temperatures, extreme pH, in the presence of organic solvents and heavy metals (Cabrera and Blamey 2018). These specific enzymes are likely to be more efficient in decomposing algal cell walls and hydrolyzing high molecular weight compounds. Therefore, direct production or heterogenous expression of these enzymes from archaea has the potential to pretreat algal biomass for down streaming processing prior to biorefinery.

### Biogas production

Algal biomass represents a promising source for renewable biogas production through anaerobic fermentation. However, the rigidity of cell walls and the high molecular weight of organic compounds pose significant challenges for biogas production (McKennedy and Sherlock 2015). To facilitate the primary microbial degradation in anaerobic digesters, archaea-derived polysaccharidases could be applied to degrade complex organic compounds into lower molecular weight organic acids. Notably, several archaeal taxa, including Methanosarcinales, Methanobacteriales, Methanomicrobiales and Methanococcales, are known to be involved in methane production (Thakur et al. 2022; Wirth et al. 2015). Bioaugmentation with the green alga (*Haematococcus pluvialis*) in anaerobic membrane reactors increased biogas production by 40% and resulted in a significant change in the relative abundance of archaea. The main methanogenesis taxa shifted from Methanothermobacter to Methanosaeta (Aydin et al. 2023). On the other hand, it is worthy noting that inhibitory compounds in algae have adverse effects on archaeal activity and limit methanogenesis. Nevertheless, there is currently little information available regarding microbial shifts during the digestion of algal biomass (Thakur et al. 2022). Improving our understanding of interactions between algae and archaea may lead to a deeper insight of the inhibitory mechanisms and inform management strategies aimed at maximizing biomethane potential.

### Wastewater treatment

**Fig. 4:**
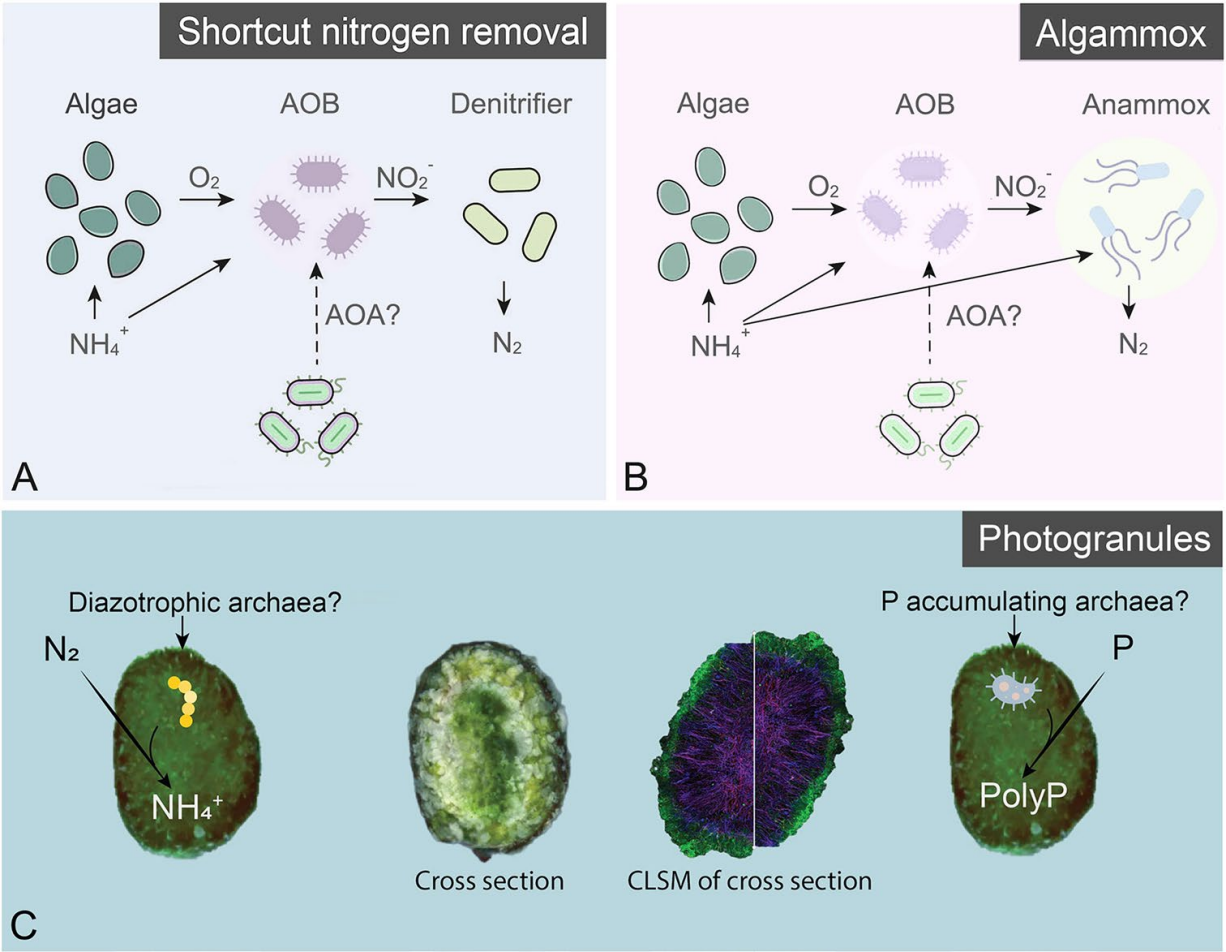
Algal–bacterial consortia are involved in **A** shortcut nitrogen removal, **B** algammox and **C** photogranules. Archaea could play a role in ammonia oxidation, nitrogen fixation and phosphate accumulation instead of bacteria. AOB and AOA denote ammonia-oxidizing bacteria and ammonia-oxidizing archaea, respectively. For the photogranules (Adapted from Trebuch et al. 2023a), the first and forth show the whole photogranules; the second shows the cross section of the photogranule and the third shows a photogranule cross section obtained by confocal laser scanning microscopy (CLSM)

As described above, archaea play a vital role in the global nitrogen cycle by performing ammonia oxidation (Francis et al. 2007). In the context of wastewater treatment, this nitrogen conversion process has recently been suggested, in combination with microalgae, as “shortcut nitrogen removal” (Wang et al. 2015) and the “Algammox” process (Manser et al. 2016; Yang et al. 2022) for the treatment of nitrogen-rich wastewaters (Fig. 4A, B). Shortcut nitrogen removal proposes a nitrification step (NH_4_^+^ to NO_2_^−^), fueled by photosynthetically produced oxygen and a subsequent denitrification step converting NO_2_^−^ to N_2_ (Wang et al. 2015). The denitrification step can occur separately in a different treatment stage or simultaneously with the nitrification step. Like the shortcut nitrogen removal process, the Algammox system is primarily directed at nitrogen removal by conversion of NH_4_^+^ to N_2_ gas. However, the Algammox process utilizes a combination of microalgae, ammonia-oxidizing organisms, and anammox bacteria in a granular form (Mukarunyana et al. 2018). Oxygen produced by microalgae drives partial nitrification to produce NO_2_^−^, which anammox bacteria use in combination with available NH_4_^+^ to produce N_2_ gas (Yang et al. 2022). Although both proposed treatment systems are described using microalgal-bacterial consortia, the potential role of ammonium oxidizing archaea in this nitrogen conversion cannot be understated. Therefore, future endeavors should conduct thorough analyses of the microbial community composition and particularly decipher the interactions of microalgae and archaea in these wastewater treatment systems.

Other technologies based on the symbiosis between microalgae and prokaryotes, such as high-rate algal ponds (HRAPs) and photogranules, have emerged as promising platforms for wastewater treatment. HRAPs and photogranules enable the removal of organic matter and nutrients with reduced or eliminated mechanical aeration, while also facilitating the capture of CO_2_ and the production of various bioproducts (Ansari et al. 2019; Solimeno et al. 2017). The most abundant archaeal taxa found in HRAPs are usually methanogens (Cantera et al. 2021; Ferro et al. 2020), where they contribute to the mineralization of organic matter by interaction with bacteria and the production of CH_4_ (Ferrera and Sánchez 2016). Photogranules are spherical aggregates composed of complex phototrophic ecosystems. Trebuch et al. (2023a) have shown that photogranules exhibit a clear stratification, with filamentous cyanobacteria forming a scaffold for other microorganisms. They have a dense shell composed of both phototrophic and non-phototrophic organisms, followed by a zone of radially aligned filamentous cyanobacteria and a dense and jumbled center. Coccoid eukaryotic algae are also present throughout the photogranule, and glycoconjugates surround the filamentous cyanobacteria. However, archaea remain poorly understood in photogranules because of their low abundance (Milferstedt et al. 2017). For instance, low-abundant sequences belonging to AOA (*Nitrosotenuis* sp.) and methanogens (*Methanofastidiosum*, *Methanobacterium*, etc.) were present in the microbial ecosystem of photogranules (Trebuch et al. 2020). Further, nitrification and denitrification are commonly described in nitrogen removal mechanisms in photogranules (Trebuch et al. 2023a) where AOA could have a profound impact. In another example, the biomass generation and wastewater treatment performance of photogranules have been successfully sustained under nitrogen limitation by means of nitrogen fixation of cyanobacteria (Trebuch et al. 2023b). Further, polyphosphate accumulating bacteria and cyanobacteria in photogranules enhanced phosphorus and chemical oxygen demand removal in wastewaters with a low N:P ratio (Trebuch et al. 2023b, c). Archaea, such as *Methanosarcina mazei*, can not only fix nitrogen but also accumulate polyphosphate (Paula et al. 2019). These archaea are worthy of further attention in future research because of their potential in the efficient functioning of photogranules and enhancement of phosphorus removal in wastewaters (Fig. 4C).

The structure of the archaeal and algal communities and their interactions have significant impacts on algal growth, wastewater treatment, and biogas upgrading. Therefore, fundamental research on algal–archaeal interactions, performance and metabolisms in wastewater treatment systems, as well as the environmental parameters that affect the growth and cooperation of both communities, is necessary to enhance the operation robustness and ensure the long-term sustainability of these biotechnologies in real-scale applications (Cantera et al. 2021).

## Conclusion and future prospective

The emergence of multi-omics has significantly expanded our knowledge of the phylogenetic and functional diversity of archaea from a variety of habitats. However, the low abundance of archaeal cells and the use of universal bacteria primers to identify archaea has led to the neglect of archaea from algal research, which consequently underestimates their significance in algal symbioses. Many fundamental questions still remain to be answered, for example, which archaeal species interact with algae and what the underlying mechanisms are? Are there any algicidal archaea? Additionally, how do they contribute to global biogeochemical cycles and global environmental change. To answer these questions, prior studies indicating positive correlations between algae and archaea can guide the targeting and isolation of potential algal-associated archaea. Cocultures of algae and archaea could be established to test the effects of archaea on algal growth. Integration of muti-omics with experimental work would greatly benefit our understanding of how algae-archaea interactions alter gene function, regulation, and metabolism. Stressors associated with global environmental change can be introduced to lab-scale systems to test the response of algae and their interaction with archaea, and how they mediate biogeochemical cycles. However, the main remaining challenge is still to translate this knowledge into practical applications including large-scale algal cultivation, algal-based bioenergy production and wastewater treatment. The recognition of the symbiotic algal–archaeal interaction may have significant implications for positive environmental impacts and for the exploitation of algae as a future energy source and other biotechnological applications.

## Data Availability

The data supporting this study are available from the corresponding author upon reasonable request.
